# Targeting HDAC/OAZ1 axis with a novel inhibitor effectively reverses cisplatin resistance in non-small cell lung cancer

**DOI:** 10.1038/s41419-019-1597-y

**Published:** 2019-05-24

**Authors:** Yuhong Sun, Xuefei Bao, Yong Ren, Lina Jia, Shenglan Zou, Jian Han, Mengyue Zhao, Mei Han, Hong Li, Qixiang Hua, Yi Fang, Jingyu Yang, Chunfu Wu, Guoliang Chen, Lihui Wang

**Affiliations:** 1 Department of Pharmacology, Shenyang Pharmaceutical University, Shenyang, China; 2 Benxi Institute of Pharmaceutical Research, Shenyang Pharmaceutical University, Shenyang, China; 3 Key Laboratory of Structure-Based Drugs Design and Discovery of Ministry of Education, Shenyang Pharmaceutical University, Shenyang, China; 4 Department of Pathology, Wuhan General Hospital of Chinese People’s Liberation Army, Wuhan, China

**Keywords:** Cancer therapeutic resistance, Non-small-cell lung cancer, Pharmacodynamics

## Abstract

Cisplatin yields significant efficacy and is generally used as a frontline therapy for non-small cell lung cancer (NSCLC). However, acquired resistance strongly limits its application. Here, we identified that a novel histone deacetylase (HDAC) inhibitor S11, with P-glycoprotein inhibitory activity, could obviously suppress cell growth in cisplatin-resistant NSCLC cell lines. In addition, S11 could increase the expression of Ac-H4 and p21, which confirmed its HDAC inhibitory action, suppress colony formation, and block cell migration of cisplatin-resistant NSCLC cells. Notably, co-treatment with S11 and cisplatin exhibited synergistically inhibitory efficacy in cisplatin-resistant NSCLC cells. Gene microarray data showed that OAZ1 was downregulated in resistant cells but upregulated after S11 treatment. Further study indicated that knockdown of OAZ1 by siRNA resulted in the decrease of sensitivity of resistant cells to cisplatin treatment and contributed to the increase of resistant cell migration. Additionally, ChIP assay data demonstrated that HDAC inhibitor S11 could increase the accumulation of Ac-H4 in OAZ1 promoter region, suggesting the direct regulation of OAZ1 by HDAC. Importantly, the combination of S11 and cisplatin overcome resistance through inhibiting HDAC activity and subsequently increasing the OAZ1 expression in preclinical model. Moreover, we observed that positive expression of HDAC1 was associated with the downregulation of OAZ1 in NSCLC patients with platinum-based treatment, and predicted drug resistance and poor prognosis. In summary, we demonstrated a role of HDAC/OAZ1 axis in cisplatin-resistant NSCLC and identified a promising compound to overcome cisplatin resistance.

## Introduction

Non-small cell lung cancer (NSCLC) is considered as one of the main cause of cancer-related death worldwide^1^. Due to advanced disease at diagnosis, chemotherapeutic approaches are irreplaceable for a majority of patients^2,3^. Cisplatin-based chemotherapy for advanced NSCLC is considered as the first-line option. However, acquired resistance inexorably develops after treatment for a period of time^4^. Several mechanisms had been reported to resulting in cisplatin resistance, including the loss copper transporter CTR1, which results in less platinum entering the cells^5^, and the increase of ERCC1, which remedies platinum-DNA damage^6^. Other mechanisms of resistance have also been identified by our and others groups, including the accumulation of cancer stem cell of NSCLC^7^, the increase of P-glycoprotein (P-gp)^8^, and the activation of survival pathway^9,10^. In spite of these advances, the against strategy for cisplatin resistance remains a clinical challenge. Therefore, it is an urgent need to further explore the underlying mechanisms of cisplatin resistance and find the mechanism-based reversing approach.

Histone deacetylases (HDACs), including 11 isoforms, contribute to the regulation of histone acetylation, which is recognized as an important epigenetic event^11^. HDACs remove acetyl groups from an acetylated lysine on a histone, resulting in the DNA wrapped by histones more tightly, subsequently affecting gene expression which is regulated by acetylation and de-acetylation. Many studies demonstrated that the aberrant overexpression of HDACs in different kinds of tumors is associated with the onset and progression of cancer^12,13^. Therefore, HDACs can be a promising target for cancer treatment. Notably, several recent studies have elucidated that HDAC inhibitors could exhibit synergistic therapeutic effects when combined with some anti-cancer agents, including DNA-damaging agents, taxanes, targeted agents, and hormonal therapies^13–16^. Furthermore, our previous study showed that continuous treatment with cisplatin resulted in the activation of HDAC, which might contribute to the accumulation of cancer stem cells and drug resistance, and the combination of HDAC inhibitor and cisplatin could synergistically inhibit tumor growth in NSCLC model^7^, suggesting HDAC inhibition might be the approach to block the development of cisplatin resistance. However, considering the P-gp-mediated drug resistance, whether HDAC inhibition could reverse the progression of cisplatin resistance in NSCLCs is still not elucidated.

Ornithine decarboxylase antizyme 1 (OAZ1) belongs to the ornithine decarboxylase (ODC) antizyme family, which mediates ODC to ubiquitin-independent proteasome degradation and suppresses the synthesis of polyamines^17^. OAZ1 was reported to display tumor-suppressor activities through affecting the cell proliferation, apoptosis, and differentiation in oral cancer cell lines and leukemia^18,19^. However, the role of OAZ1 in lung cancer, especially in drug resistance, and its regulation mechanism are still not known.

Here, we identified dual-targeting inhibitor S11, which simultaneously inhibited HDAC and P-gp. Further study showed that S11 could obviously suppress cell growth, colony formation, and cell migration of cisplatin-resistant NSCLC cells. Importantly, co-treatment with S11 and cisplatin exhibited synergistically inhibitory efficacy in cisplatin-resistant in vitro and in vivo NSCLC models. Gene microarray data demonstrated that OAZ1 was the key gene in mediating this reverse process. Gene manipulation confirmed the important role of OAZ1 in drug resistance and cell migration, and ChIP assay explored the epigenetic regulation of OAZ1 by HDAC. Notably, we observed that overexpressed HDAC1 was associated with the downregulation of OAZ1 in advanced NSCLC patients with cisplatin treatment, and predicted chemotherapy resistance and bad outcome. Our finding not only identified a novel dual-targeting compound to reverse acquired resistance to cisplatin in NSCLC, but also demonstrated that HDAC1 combined with OAZ1 might be a valuable response biomarker for cisplatin treatment.

## Results

### The discovery of S11

Belinostat (PXD101), an HDACs inhibitor, was approved for the treatment of refractory peripheral T-cell lymphoma^20^. It has been reported to circumvent the resistance in NSCLC through inhibition of both ABCC2 and DNA repair-mediated mechanisms^21^. Meanwhile, in our previous work, SNOH-3 derived from belinostat could effectively reverse paclitaxel resistance in NSCLC via multiple mechanisms^14^.

**Scheme 1:**
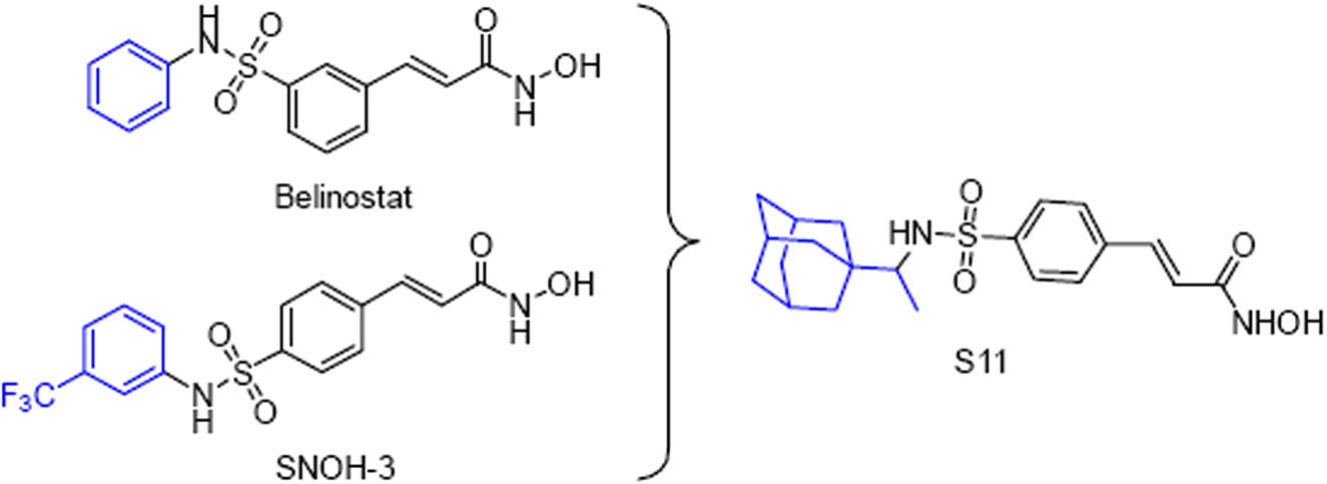
The design rationale for S11

A widely accepted pharmacophore model for HDAC inhibitors consists of a metal-binding head group, a linker domain, and a cap group, which is a common group for modification^22^. Although many derivatives of belinostat were obtained, the caps of them were almost all aromatic groups. Therefore, non-planar adamantane moieties were employed to replace the terminal phenyl group of belinostat, and the substituted pattern of phenyl linker is changed from meta-position to para-position (Scheme 1).

### Synthesis of S11

**Scheme 2:**
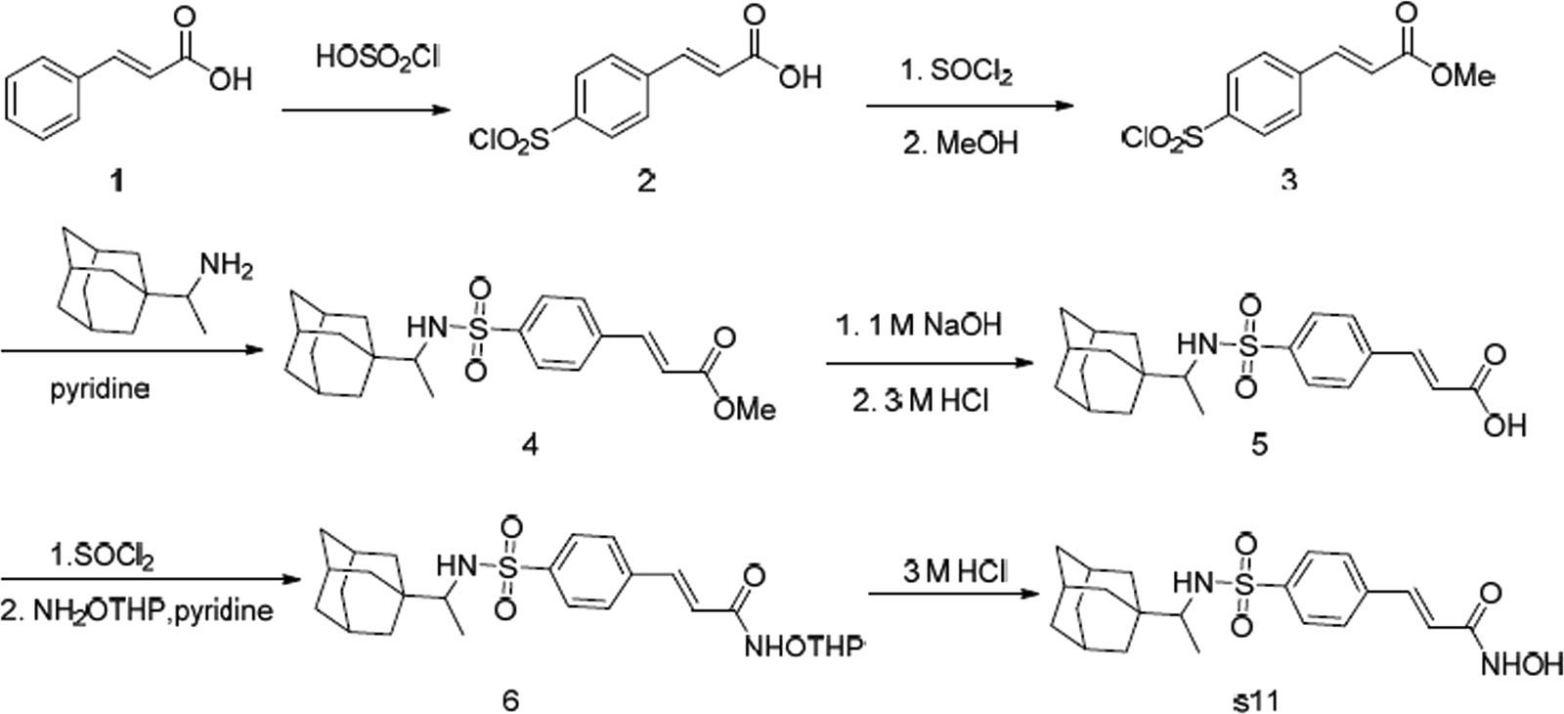
Synthesis of S11

The route adopted for the preparation of S11 is depicted in Scheme 2. Compound 3, obtained according to our previous work^23^, was reacted with the adamantine-based amine to afford sulfonamide derivative 4, which was transformed into the cinnamic acid 5. Next, cinnamic acid 5 was reacted with sulfoxide chloride and then hydroxylamine protected by Tetarhydropyran (THP) to afford the amide derivative 6, and the *N*-hydroxypropenamide S11 was obtained via deprotection. The structure of S11 was characterized by 1H-NMR (nuclear magnetic resonance), mass spectrometry (MS), and HRMS High Resolution Mass Spectrometry (HRMS).

### Molecular docking

**Fig. 1:**
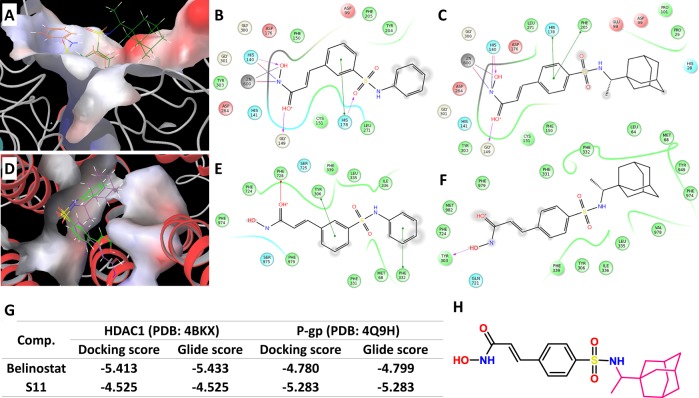
Molecular docking of compound S11 and belinostat (PXD101). **a** Comparative binding modes of compound S11 (purple) and belinostat (green) in the active site of HDAC1; **b** 2D binding model of belinostat into the HDAC1; **c** 2D binding model of S11 into the HDAC2; **d** comparative binding modes of compound S11 (purple) and belinostat (green) in the active site of P-gp; **e** 2D binding model of belinostat into the P-gp; **f** 2D binding model of S11 into the P-gp; **g** docking score and Glide score of belinostat and S11; **h** the structure of S11

Molecular docking showed that the combination pattern of S11 with HDAC1 (PDB ID: 4BKX) was similar to that of belinostat. Compared with belinostat, the sulfonyl group of S11 could not form hydrogen bonds with HDAC1 via HIS178, which could, to a certain extent, explain the weaker binding capacity of S11 (Fig. 1a–c). Barreto et al.^24^ solved an X-ray crystal structure of mouse P-gp without nucleotide or drug substrate (PDB ID: 4Q9H), which was employed for molecular docking in this study. The docking study revealed that belinostat exhibited moderate binding affinity with a Glide score (GScore) of −4.799, while the binding affinity of compound S11 was stronger with a GScore of −5.283. The adamantine group of S11 inserted deeply into the hydrophobic pocket surrounded by Phe332, Met68, Tyr949, and Val978, whereas the terminal phenyl group of belinostat was relatively far from this hydrophobic pocket, which would weaken the hydrophobic interaction between the small ligand and target protein, thus reducing the binding affinity between belinostat and P-gp (Fig. 1d–f).

### S11 inhibited the activities of HDAC and P-gp

**Fig. 2:**
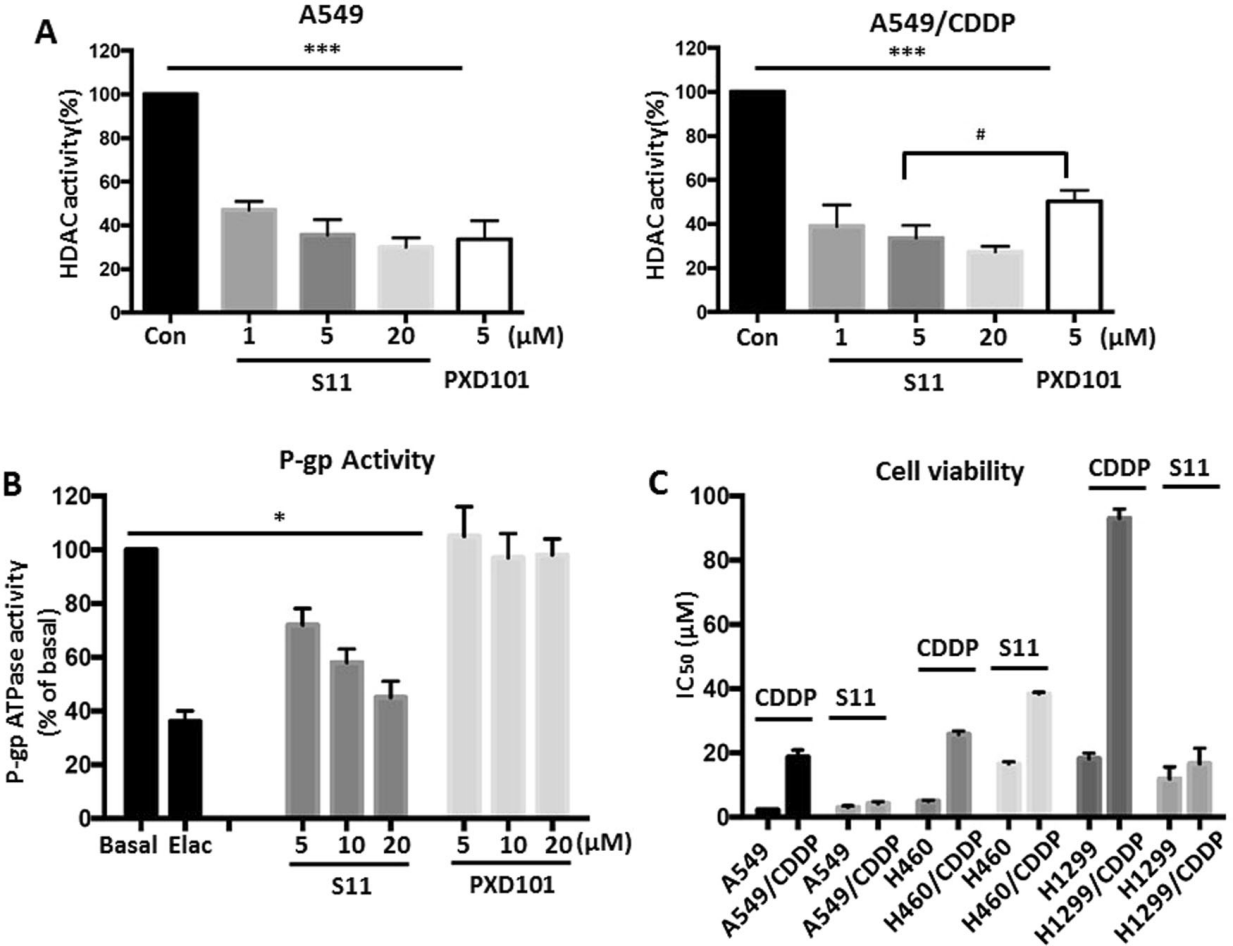
The inhibitory action of S11 on HDAC, P-gp, and cisplatin-resistant NSCLC cells. **a** HDAC activity was detected by microplate reader in S11- and DMSO-treated A549 and A549/CDDP cells. PXD101 was used as positive control. **b** The P-gp ATPase activity in vitro was measured by Pgp Glo assay system. Elacridar was used as positive control. **c** The cell viability of A549, A549/CDDP, NCI-H460, NCI-H460/CDDP, NCI-H1299, and NCI-H1299/CDDP cells after treatment with different concentrations of S11 and CDDP for 48 h

To confirm the molecular docking data, we investigated the effect of S11 on the activities of HDAC and P-gp using cell-based experiments. As shown in Fig. 2a, S11 could significantly inhibit the HDAC activity in a concentration-dependent manner in A549 cells. Compared with positive control compound PXD101, S11 displayed a similar efficacy at the same concentration. Notably, we found that S11 exhibited an enhanced inhibitory action as compared with PXD101 in cisplatin-resistant A549 cells (Fig. 2a), suggesting a special inhibitory action of S11 in resistant cells. In addition, NCI-H460/CDDP cells showed same results, which confirmed this action (see Supplementary Fig. 1A). Interestingly, the expression of HDAC1, the main isoform of HDACs, in A549/CDDP and NCI-H460/CDDP cells did not decrease after the treatment with S11 or PXD101, suggesting that S11 inhibited HDAC activity but did not affect the HDAC1 expression (see Supplementary Fig. 1B, C). As compared with parental cells, A549/CDDP cells showed an increasing expression in P-gp level (see Supplementary Fig. 2A). Thus, we next measured the effect of S11 on P-gp activity using P-gp Glo assay in vitro. As indicated in Fig. 2b, P-gp inhibitor elacridar displayed a significant inhibition compared with control group. Importantly, S11 treatment resulted in a concentration-dependent inhibition of P-gp activity. In contrast to S11, PXD101 could not affect P-gp activity at the same concentrations with S11. Furthermore, Rh123 retention assays were also used to assess P-gp function in vitro in A549/CDDP and NCI-H460/CDDP cells and showed similar results with P-gp Glo assay (see Supplementary Fig. 2C). These results indicated that S11 can reduce drug efflux in A549/CDDP and NCI-H460/CDDP cells and inhibit the transmembrane pumping function of P-gp.

To further explore the effect of S11 on cisplatin-resistant cells, we detected the cell viability of three paired parental and cisplatin-resistant NSCLC cells after treatment with S11. As shown in Fig. 2c, A549/CDDP, NCI-H460/CDDP, and NCI-H1299/CDDP cells showed a resistance index (RI) of 8.46, 5.18, and 5.11, respectively. However, S11 treatment not only displayed a lower IC_50_, especially in A549/CDDP and NCI-H1299/CDDP cells, but also exhibited decreasing RI values (1.38 for A549/CDDP vs. A549, 2.32 for H460/CDDP vs. H460, 1.39 for H1299/CDDP vs. H1299), demonstrating a reverse drug-resistant characteristic of S11 in NSCLC cells. On the contrary, PXD101 treatment exhibited higher IC_50_ and RI values, especially in A549 and NCI-H460 resistant cell lines (see Supplementary Fig. 2B), suggesting that the potential of P-gp inhibition might be an important characteristic for HDAC inhibitor against cisplatin resistance.

### S11 retarded the malignant behavior and synergistically combined with cisplatin-suppressed cell growth in cisplatin-resistant NSCLC cells

**Fig. 3:**
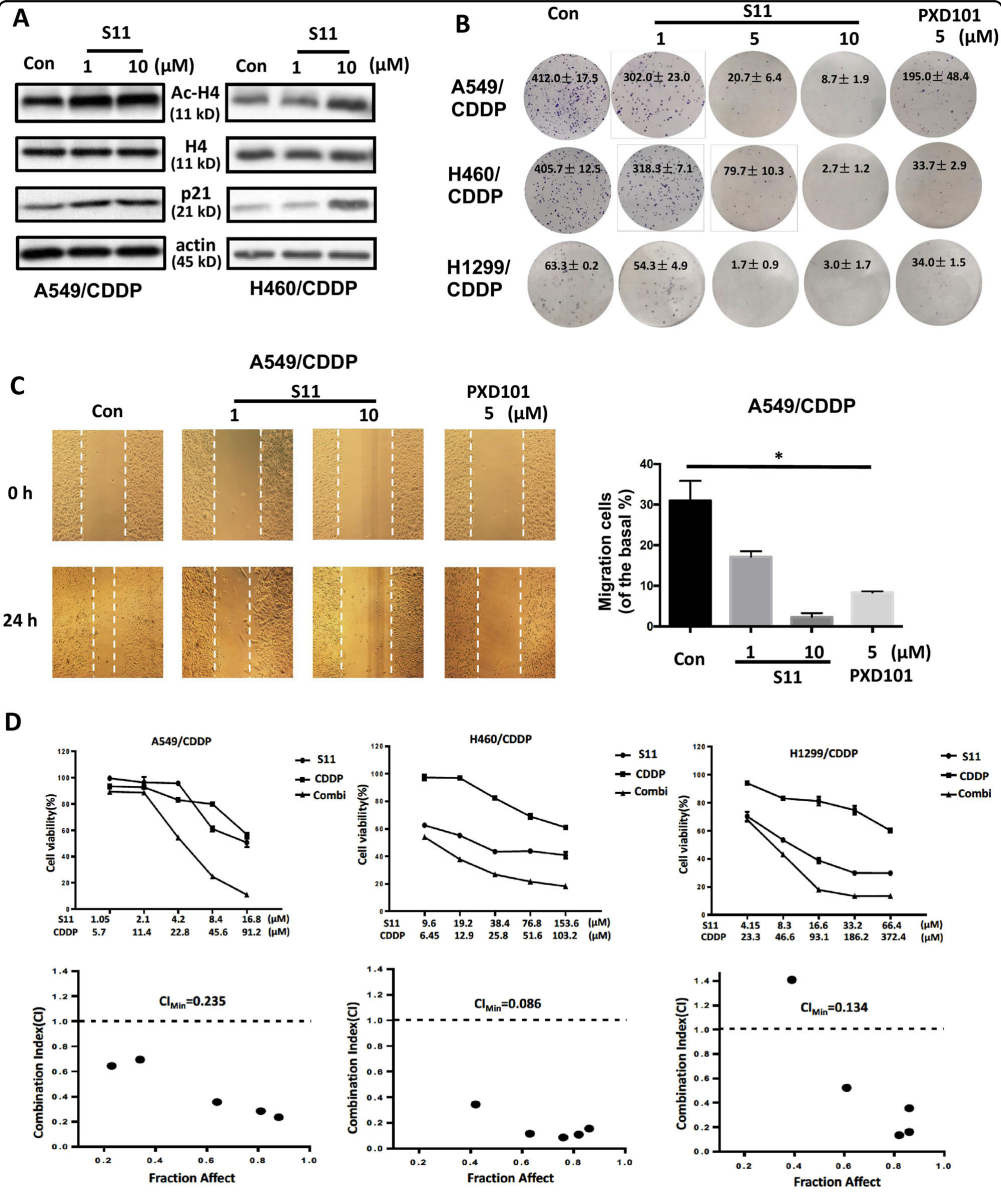
The effects of S11 on HDAC substrates, cell growth, migration, and synergistic action in cisplatin-resistant NSCLC cells. **a** Ac-H4 and p21 were measured in S11- and PXD101-treated NCI-H460/CDDP and A549/CDDP cell lines. H4 and β-actin expression was used as a loading control. **b** The colony formation assay was accessed in S11- and PXD101-treated cisplatin resistant NSCLC cells. **c** The migration ability was accessed in S11- and PXD101-treated A549/CDDP cell line by scratch-wound healing assay. **d** The growth curve of NCI-H460 cells and A549 cells after treatment with S11, CDDP, and the combination of S11 and CDDP. Analysis of the combination of S11 and CDDP in both cell lines. The cells were treated for 48 h using increasing concentrations of S11 and CDDP, either alone or in a fixed ratio. The resultant data were analyzed using Calcusyn program, and graphs from the averaged results of three independent experiments are shown. All error bars are s.e.m. *P < 0.05, compared with control

To further investigate the anti-tumor potential of S11, we detected the effect of S11 on HDAC substrates, colony formation, and migration ability. Western blot data showed S11 treatment led to the increase of Ac-H4 and its substrate p21, a tumor-suppressor protein, in A549/CDDP and NCI-H460/CDDP cells (see Fig. 3a). Moreover, S11 also reduced the colony formation number in a concentration-dependent manner in three cisplatin-resistant NSCLC cell lines. Notably, compared to PXD101, S11 exhibited an enhanced inhibitory potential at the same concentration (5 μM) in A549/CDDP and NCI-H1299/CDDP cell lines (see Fig. 3b). In addition, S11 also displayed the migration inhibitory ability when treated in A549/CDDP cells (see Fig. 3c). Similarly, S11 also suppressed cell migration in NCI-H460/CDDP cells (see Supplementary Fig. 3A). Next, we explored the effect of S11 combined with cisplatin on cell growth in three cisplatin-resistant NSCLC cells. As shown in Fig. 3d, the combination of S11 and cisplatin resulted in an obviously synergistic inhibition in three cisplatin-resistant NSCLC cell lines. The maximal synergistic efficacy was obtained in NCI-H460/CDDP cells, with the CI_Min_ value 0.086. The synergistic effect of S11 combined with CDDP was confirmed by apoptosis assay, which showed that S11 combined with cisplatin could obviously induce cell apoptosis in three cisplatin-resistant NSCLC cells (see Supplementary Fig. 3B).

### S11 inhibited the growth of cisplatin-resistant cells through upregulating OAZ1

**Fig. 4:**
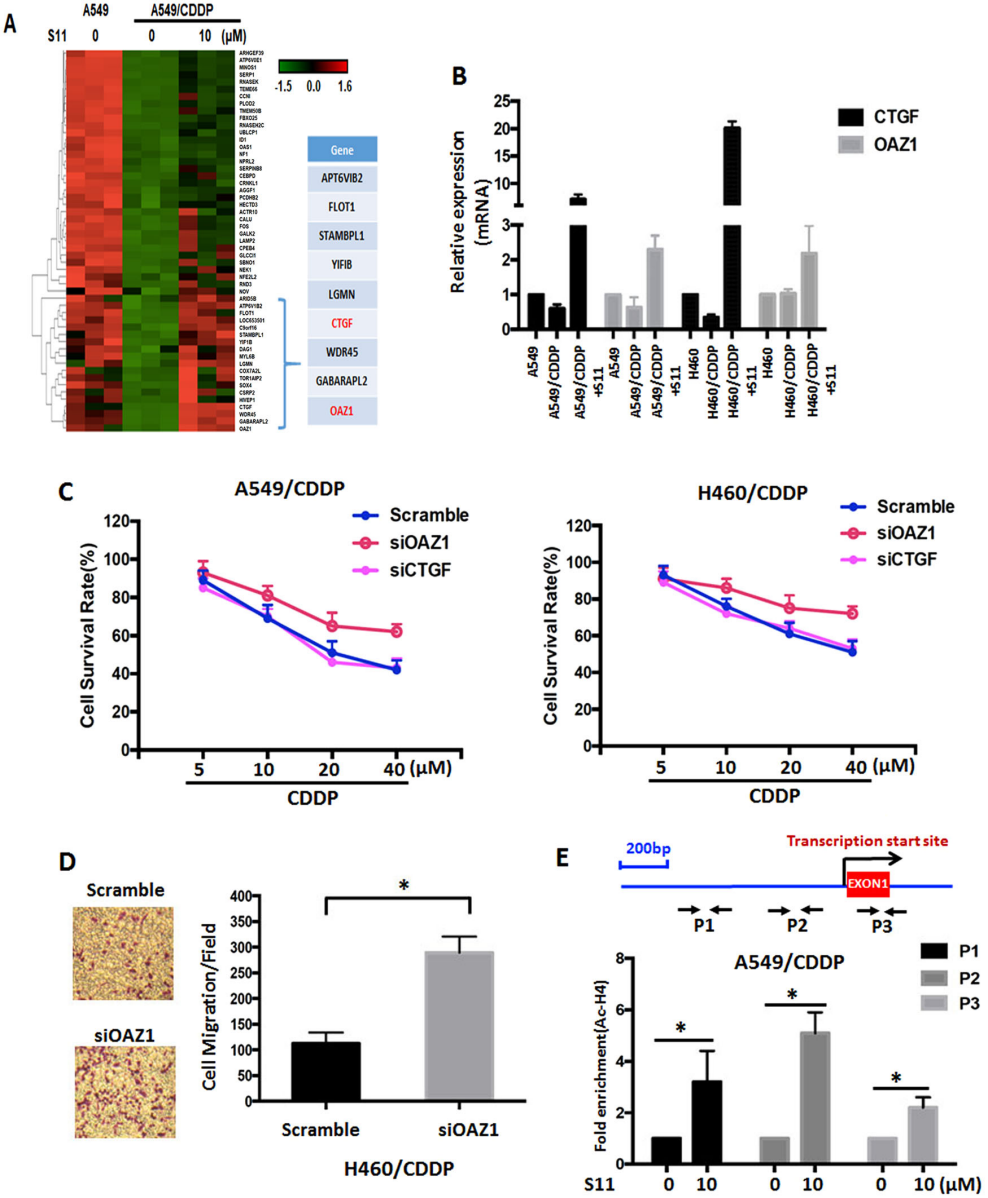
The identification and role of OAZ1 in cisplatin-resistant NSCLC cells. **a** Heat map depicting the differential gene expression among A549, A549/CDDP, and S11-treated A549/CDDP cells. Pink and blue indicate high and low mRNA expression levels, respectively. **b** The expression levels of CTGF and OAZ1 were detected by real-time PCR in S11-treated and control A549/CDDP and NCI-H460/CDDP cells, and their parental cells. **c** The sensitivity to CDDP was accessed in A549/CDDP and NCI-H460/CDDP cells after knockdown of OAZ1 and CTGF by siRNA (100 nM) for 48 h. **d** The migration ability was accessed in NCI-H460/CDDP cells after knockdown of OAZ1 by siRNA (100 nM) for 48 h. **e** ChIP assays confirmed the binding of Ac-H4 to the promoter regions upstream of OAZ1 gene. All error bars are s.e.m. *P < 0.05, compared with Scramble or control

To further elucidate the mechanism of the reversing effect of S11 on cisplatin-resistant cells, we performed gene microarray analysis. In view of the characteristics of HDAC inhibitors, which usually result in chromatin loose and then mediate the upregulation of target gene^12^, we focus on the changed genes that were downregulated in resistant cells, meanwhile were upregulated after S11 treatment. As shown in Fig. 4a, there were nine genes complied with the standard. Among the nine genes, two genes, CTGF and OAZ1, were reported to be associated with tumor biology^18,24^. Thus, we selected both genes in the next study. Real-time PCR data confirmed the gene microarray data, both genes were downregulated in A549/CDDP cells and upregulated after treatment with S11 (see Fig. 4b). Similar results were obtained in NCI-H460/CDDP cells. Next, we explored the role of both genes in cisplatin resistance using specific siRNA interference. As shown in Fig. 4c and Supplementary Fig. 4A, knockdown of OAZ1 by specific siRNA could decrease the sensitivity of both A549/CDDP and NCI-H460/CDDP cells to cisplatin. In contrast to OAZ1 knockdown, the silence of CTGF could not affect the sensitivity of both resistant cell lines to cisplatin (Fig. 4c and Supplementary Fig. 4B). The above data suggested OAZ1 might play a key role in cisplatin resistance. In addition, we also found that knockdown of OAZ1 led to the increase of cell migration potential in NCI-H460/CDDP cells (Fig. 4d), which further confirmed the role of OAZ1 in cisplatin resistance.

To further explore the regulation relationship between HDAC and OAZ1, we measured the binding ability of Ac-H4, marker of histone acetylation, in A549/CDDP cells. As shown in Fig. 4e, inhibiting HDAC by S11 contributed to increase in the extent to which Ac-H4 was enriched in the indicated three promoter regions (P1, P2, and P3), suggesting that HDAC plays a role in OAZ1 transcriptional regulation in cisplatin-resistant cells. The above results demonstrated that S11 inhibited the malignant behavior of cisplatin-resistant NSCLC cells through HDAC/OAZ1 axis.

### S11 combined with cisplatin abrogated cisplatin resistance in vivo

**Fig. 5:**
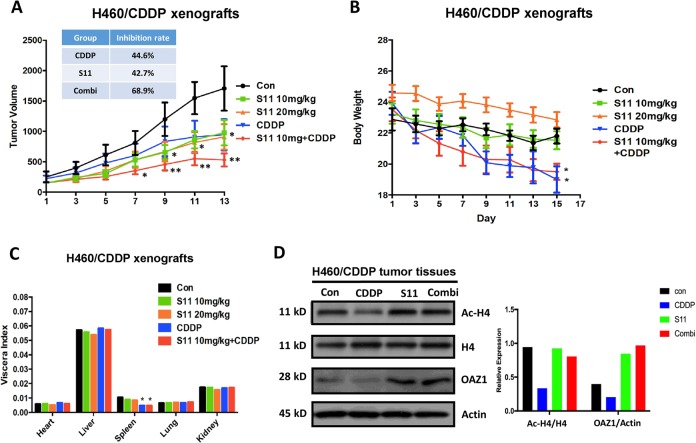
Effects of S11 and/or CDDP on tumor growth, body weight, organ index, and biomarkers in an CDDP-resistant xenograft model. **a** Tumor volumes, **b** body weights, and **c** organ indexes were measured in NCI-H460/CDDP xenografts treated with S11, CDDP, or the combination of S11 and CDDP. **d** Ac-H4, H4, and OAZ1 expression levels were measured in NCI-H460/CDDP xenograft tumor tissues. β-actin was used as a loading control. *P *<* 0.05, **P < 0.01, compared to control

We next evaluated the application potential of the combination of S11 and cisplatin as a therapeutic approach in vivo. BALB/c mice bearing NCI-H460/CDDP xenografts were treated with cisplatin alone, S11 alone (10 mg/kg and 20 mg/kg), or dual combinations (S11 10 mg/kg plus cisplatin). The xenografts treated with cisplatin showed a relatively slow and weak inhibition, which verifies a resistant characteristic of mice tumor model (Fig. 5a). Compared to cisplatin, S11 at both dosages displayed an earlier inhibition during the course of the experiment, with administration for 9 days showing an obvious inhibition (Fig. 5a). Remarkably, mice treated with the dual combination displayed an earlier efficacy, and the robust inhibition of tumor growth with an inhibition rate of 68.9%, compared with single-treatment groups, therefore mirroring the in vitro results (Fig. 5a). In addition, we also evaluated the toxicity after treatment with S11. As shown in Fig. 5b, c, single treatment with S11 could not result in obvious changes in body weight and organ index as compared to the control group. In contrast to S11, cisplatin treatment would lead to the loss of body weight and decrease in spleen index. Importantly, the combination of S11 and cisplatin could not enhance the cisplatin-triggered toxicity, suggesting the acceptable toxicity profile of S11 as a single or combined treatment approach.

Furthermore, we also examined the Ac-H4 and OAZ-1 expression levels in tumor tissue samples from the four groups (Fig. 5d). Consistent with the above in vitro results, these results showed that administration of S11 could increase the expressions of Ac-H4 and OAZ-1. On the contrary, single treatment with cisplatin led to the decrease of Ac-H4 and OAZ-1. Notably, similar to S11 single treatment, the combination of S11 and cisplatin resulted in an increase of Ac-H4 and OAZ1. In addition, we also found that CDDP single treatment could induce P-gp expression, whereas combination treatment could reverse this effect (see Supplementary Fig. 2D). Taken together, these findings demonstrated that ablation of HDAC could sensitize cisplatin-resistant cells in vivo by upregulating OAZ1.

### Cases with increased HDAC1 and decreased OAZ1 expressions predicted to bad outcome of NSCLC patients treated with platinum

**Fig. 6:**
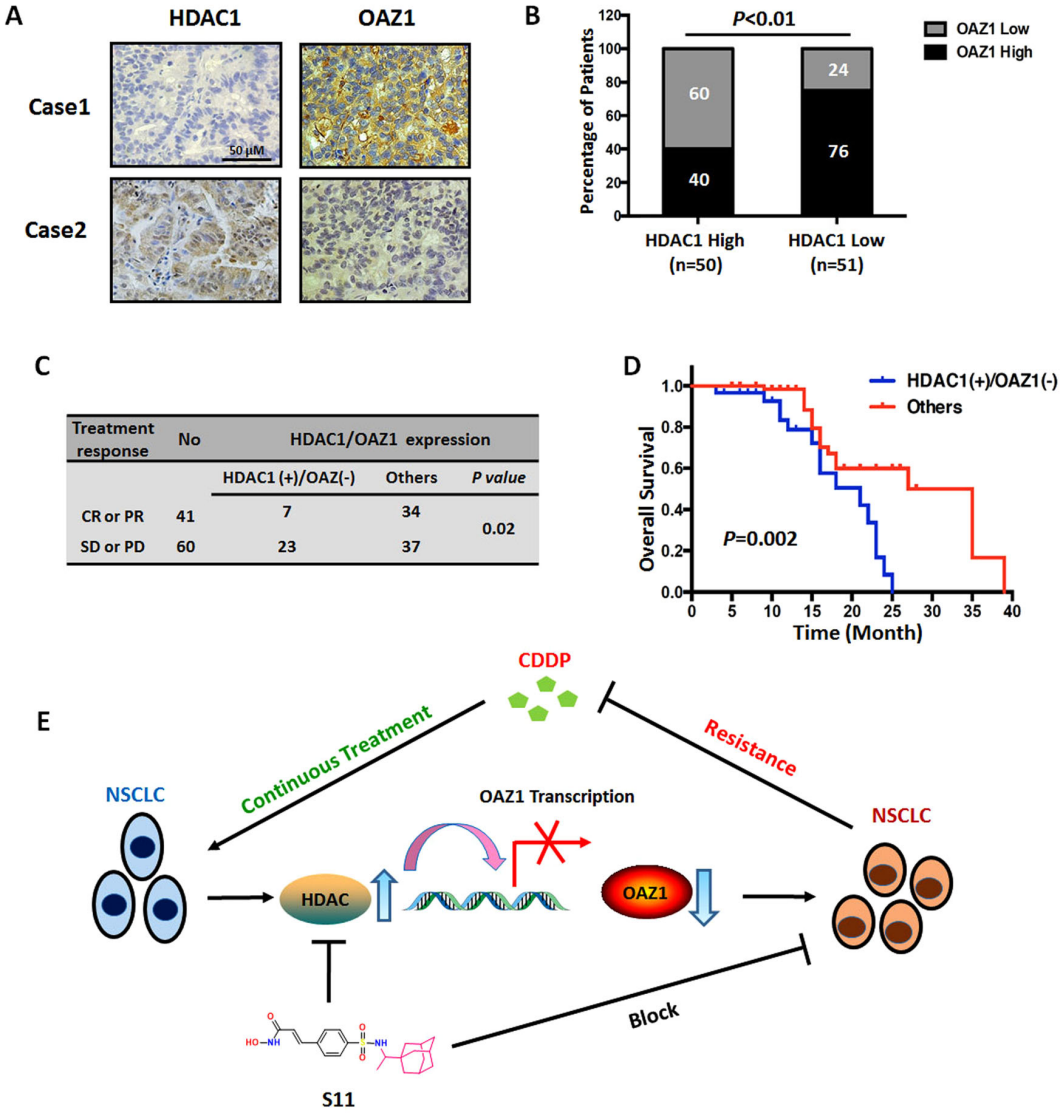
Measurement of HDAC1 and OAZ1 expression in clinical NSCLCs treated with platinum-based chemotherapy. **a** Representative images of HDAC1 and OAZ1 expression in NSCLC tissues. **b** The correlation between HDAC1 expression and OAZ1 expression in NSCLC tissue specimens. The combination of high HDAC1 expression and low OAZ1 expression was correlated with a bad response **(c)** and overall survival **(d)** in patients with platinum-based chemotherapy. **e** Schematic of the mechanism by which HDAC activation results in the downregulation of OAZ1 and induces cisplatin resistance in NSCLCs and reversing resistance by novel HDAC inhibitor S11

To confirm the above-mentioned relationship between HDAC and OAZ1, we next investigated the expressions of HDAC1, the main isoform of HDACs, and OAZ1 using immunohistochemistry method in tissue specimens from 101 platinum-treated NSCLC patients (Fig. 6a). Our data showed that 76% of the patients with higher OAZ1 expression (*n* = 39) were in the low HDAC1 expression group (*n* = 51), whereas 60% of the patients with lower OAZ1 expression (*n* = 30) were in the high HDAC1 expression group (*n* = 50), suggesting that OAZ1 expression was negatively associated with HDAC1 expression in platinum-treated NSCLC cases (*P* < 0.05, Fig. 6b). Importantly, the opposite expression pattern (higher HDAC1 and lower OAZ1) correlated with bad treatment response and poor overall survival than other groups in lung cancer (Fig. 6c, d). It should be noted that the PROGgeneV2 database (GSE30219, Supplementary Fig. 4C) also showed that this expression pattern was obviously correlated with poor prognosis. In summary, HDAC1 along with OAZ1 may be used as a predictive biomarker for platinum-treated response and their alterations may be correlated with platinum resistance and prognosis.

## Discussion

Cisplatin-based chemotherapeutics are widely used against NSCLC; thus, preventing or blocking cisplatin resistance is considered as a crucial clinical issue in the treatment of NSCLC^3^. In the present study, we identified a dual-targeting compound, S11, which could inhibit HDAC function and block P-gp activity. Notably, S11 treatment resulted in obvious suppression of cell growth in cisplatin-resistant NSCLC cells and it synergistically enhanced the sensitivity of resistant cells to cisplatin. Mechanistically, S11 could upregulate OAZ1, a candidate tumor-suppressor gene, through inhibiting HDAC activity. Importantly, the combination of S11 and cisplatin exhibited an enhanced antitumor efficacy in cisplatin-resistant NSCLC xenograft model. Clinical immunohistochemistry indicated that tumors with higher HDAC1 accompanied with lower OAZ1 expression may show a poor response to platinum treatment and poorer prognosis than tumors with other expression patterns in patients with NSCLC. Our findings provide us with a novel perspective regarding cisplatin resistance and indicate that the novel dual-targeting compound S11 may show promise as a treatment for patients with cisplatin-resistant NSCLC.

P-gp is considered as one of the most important drug-resistant protein, which mediates its substrate efflux from the cell^25^. In fact, numerous reports indicated that cisplatin did not belong to the P-gp substrate, suggesting that P-gp inhibition might be not correlated with the cisplatin resistance^26^. However, in our present study, we found that S11, having P-gp inhibitory activity, displayed an enhanced HDAC suppression and resistance reversing activities as compared to the HDAC inhibitor PXD101, suggesting that P-gp might be involved into cisplatin resistance in NSCLCs. This viewpoint could be supported by the other group studies. Gibalová et al. demonstrated that the P-gp-induced changes in cell regulatory pathways, which result in a partial loss of cisplatin sensitivity of P-gp, are independent of its drug efflux activity^27^. In addition, several studies indicated that P-gp protein was overexpressed in cisplatin-resistant cells^28,29^, which further confirmed the role of P-gp in cisplatin resistance. Therefore, the P-gp inhibitor, as an adjuvant drug, might be a potential approach to reverse cisplatin resistance in NSCLC.

OAZ1 plays an important role in the regulation of polyamine biosynthesis through binding and inhibiting ODC, which is a key enzyme during polyamine biosynthesis^17^. Several reports have indicated that OAZ1 was associated with malignant behaviors, such as tumor growth, apoptosis, and differentiation in various cancer types. Wang and Jiang found that OAZ1 overexpression resulted in the inhibition of cell proliferation and induction of cell differentiation through upregulating LOR, a differentiation biomarker, in human oral cancer cells^18^. In addition, Wu et al. demonstrated that OAZ1 was downregulated in chronic myeloid leukemia cases, and overexpression led to cell differentiation^19^. The above results verified that OAZ1 gene might be a tumor-suppressor gene. Our results also demonstrated that OAZ1 expression level was downregulated in NSCLC cisplatin-resistant cells, but was upregulated in HDAC inhibitor S11-treated resistant cells. Moreover, knocking down OAZ1 expression conferred the decrease in sensitivity to cisplatin and increase in cell migration capability in cisplatin-resistant NSCLC cells, indicating that OAZ1 plays a tumor-suppressor role in NSCLC. However, the detailed function and the underlying mechanisms need be further clarified.

In general, the activation of tumor-suppressor gene is important to block tumor development and progression, and also could be considered as a promising therapeutic strategy. Here, we found that S11, an HDAC inhibitor, could activate OAZ1 by enhancing Ac-H4 accumulation in the promoter region of OAZ1, which not only indicates a direct transcription regulation mechanism between HDAC and OAZ1, but also provides a candidate approach to restore OAZ1. Furthermore, our clinical study also demonstrated the regulation relationship between HDAC1 and OAZ1, and verified the potential value of HDAC1 combined with OAZ1 as a therapeutic and prognostic biomarker.

The resistance to cisplatin in NSCLC usually leads to treatment failure. In the present study, our results, that HDAC inhibitor S11 inhibited the tumor growth of cisplatin-resistant NSCLC cells and malignant behavior through upregulating OAZ1, prompted us to evaluate the effectiveness of combination therapy as an approach for cisplatin-resistant NSCLC. We found that administering S11 in combination with cisplatin had an obvious effect on tumor growth in preclinical models and without enhancing cisplatin toxicity. Notably, a recent study showed that SAHA, an FDA-approved HDACs inhibitor, improved the efficacy of platinum-based chemotherapy in patients with advanced NSCLC^30^. Also, several studies have shown that the activity of HDACs or their expressions were increasing in chemotherapeutic/TKI resistant NSCLC cells^14,31^, and the combination of HDACs inhibitor with chemotherapeutic/TKI agents has been demonstrated to be against drug resistance^14,15,32^. Given that HDAC inhibitor owns acceptable toxicity profile and targetable value, treatments targeting the HDAC may be useful for reversing drug resistance in NSCLC.

Taken together, a common and inevitable phenomenon in clinical oncology is acquired drug resistance. The present study demonstrated that elevated HDAC activity and increased P-gp expression in NSCLC may induce resistance to treatment with cisplatin, indicating that both proteins, especially HDAC, may be useful indicators of cisplatin resistance in NSCLC. The increase in HDAC activity would contribute to the epigenetic-mediated downregulation of OAZ1, a candidate tumor-suppressor gene, and thus results in drug resistance (Fig. 6e). Furthermore, we identified that a dual-targeting (HDAC/P-gp) compound, S11, could inhibit the malignant behavior of cisplatin-resistant cells and reverse cisplatin resistance in in vitro and in vivo models. Therefore, we not only illustrated that epigenetic regulation axis HDAC/OAZ1 are responsible for cisplatin resistance in NSCLC but also provided a feasible therapeutic approach against cisplatin resistance.

## Materials and methods

### Chemistry

X-4 apparatus was employed to determine the melting points and was uncorrected. The NMR spectra and electrospray ionization mass spectrometry (ESI-MS) analyses were recorded on Bruker Ascend 400 (Billerica, MA, USA) and Agilent 1100 Series MSD Trap SL (Santa Clara, CA, USA), respectively. Commercially available reagents and solvents were used without further purification.

### Molecular docking study

The molecular docking was conducted using the GLIDE program (version 10.2, Schrodinger, LLC, New York, 2015). The murine P-gp structure (ID: 4Q9H) and the crystal structure of human HDAC1 (ID: 4BKX) were obtained from the Protein Data Bank (www.pdb.org), and the Protein Preparation Wizard module was applied for the protein structure preparation. The ligands were minimized by means of the LigPrep module. The receptor grid file, determining the position and size of the active site, was generated according literatures^33,34^, which reported the key amino acids forming the binding pocket. SP mode was employed to perform Glide docking with default protocols. After scoring the docked complexes via the GScoring function, the binding mode and critical interactions with the key amino acids of these complexes with higher GScores were analyzed.

### Cell lines and cell culture

A549, NCI-H460, and NCI-H1299 human lung adenocarcinoma cell lines were obtained from American Type Culture Collection (ATCC; Manassas, VA, USA). The cells were cultured in RPMI-1640 medium (Gibco, Grand Island, NY, USA) supplemented with 10% fetal bovine serum (FBS; Gibco) and were maintained at 37 °C in a humidified incubator with 5% CO_2_. The resistant cells were obtained after treatment of parental cell lines with cisplatin (J&K Scientific Ltd., Beijing, China) at increasing concentrations (0.5, 2, or 5 μM/per month) for 3 months.

### Compounds and reagents

Belinostat (PXD101) and elacridar were purchased from MedChemExpress, USA. These agents were dissolved in DMSO to 100 mM and stored at −20 °C. Before treatment, the stock solution is diluted to different concentrations. The final concentration of DMSO in cultures is 0.1% (v/v) or less. MTT (3-(4,5-dimethylthiazol-2-yl)-2,5-diphenyl tetrazolium bromide) was obtained from Sigma, USA. The primary antibodies against OAZ1 and P-gp were obtained from Abcam (Cambridge, MA). The primary antibodies against acetylated Histone 4(Ac-H4) were purchased from Millipore, USA. The primary antibodies against HDAC1, Histone 4, p21, and β-actin were obtained from Cell Signaling Technology (Danvers, MA). The OAZ1 Silencer Select Validated siRNA was obtained from Life Technologies, USA.

### Patients and chemotherapy

A total of 101 patients with advanced NSCLC (stage IIIB and stage IV) were enrolled between January 2004 and June 2016 from Wuhan General Hospital of Guangzhou Command (Wuhan, PR, China). The enrolled patients' information and chemotherapy processes are provided in the Supplementary Materials and Methods.

### Immunohistochemistry

A tissue microarray (TMA) was constructed (in collaboration with the Shanghai Biochip Company Ltd.) as described previously^14^. The primary immunostaining antibody concentrations were 1:200 dilution for HDAC1 and 1:50 dilution for OAZ1. The evaluation of both the intensity of immunohistochemistry staining and the proportion of positively stained epithelial cells was previously described^14^.

### Cell viability assay

The in vitro cell viability was determined by MTT assay. The seeded cells (1 × 10^5^ cells/ml) were treated with various concentrations of agents for 48 or 72 h. The detailed procedure is provided in the Supplementary Materials and Methods.

### HDAC activity assay

The HDAC assay was conducted with an HDAC fluorescent activity assay kit (Biovision, USA) as our previous report^14^. The A549 and A549/CDDP cells were treated with different concentrations of S11 or belinostat (PXD101) for 24 h before assays. The detailed procedure is provided in the Supplementary Materials and Methods. HDAC activity is shown as the means ± s.e.m of three determinants.

### P-gp activity assay

P-gp activity was determined by the Pgp Glo assay systems (Promega) following the user protocol provided by the manufacturer. The detailed experimental and analysis processes are provided in the Supplementary Materials and Methods.

### The Rhodamine 123 efflux assay of P-gp function

A549/CDDP and NCI-460/CDDP cells were incubated with S11 or PXD101 (5 μM) for 24 h. The detailed procedure is provided in the Supplementary Materials and Methods.

### Western blot analysis

About 1 × 10^7^ cells were gathered after pre-treatment for the indicated time periods as described previously. Western blotting was performed as previously described^14^. The detail procedure is provided in the Supplementary Materials and Methods. The results were normalized to the internal control β-actin.

### Clonogenic assay

The cells were treated for 48 h at various concentrations. Two-thousand cells were then plated in 100-mm dishes for 14 days. The cells were fixed with 4% formaldehyde for 10 min, stained with 0.5% crystal violet for 30 min, and the number of colonies was counted.

### Scratch-wound healing recovery assays

Wound healing assay was used to detect cell migration ability. The detailed experimental and analysis processes are provided in the Supplementary Materials and Methods.

### Determination of combination index

A549/CDDP, NCI-H460/CDDP, and NCI-H1299/CDDP cells were administered with various concentrations of single S11, CDDP, or their combination. MTT assay was used to measure cell viability. The nature of the drug interaction was analyzed using the combination index (CI) based on the method of Chou and Talalay. A CI value <0.90 means synergism; a CI value between 0.90 and 1.10 shows additive; and a CI value >1.10 indicates antagonism. The data were analyzed by the Calcusyn software (Biosoft, Oxford, UK).

### RNA sequencing and gene expression analysis

Total RNAs of A549, A549/CDDP, and A549/CDDP, treated with S11, cells were isolated by using RNeasy Mini Kit (Qiagen, Valencia, CA, USA) as described in the product introduction. The Affymetrix GeneChip PrimeView Human Gene Expression Arrays were used to detect gene expression. The quantitative RT-PCR (qRT-PCR) was used to confirm the changed gene expression. The sequences of qPCR primers are listed in Supplementary Table 1.

### Transwell assay

NCI-H460/CDDP migration capacity was tested by Corning transwell assay, according to the manufacturer’ s instructions. The detailed experimental and analysis processes are provided in the Supplementary Materials and Methods.

### Chromatin immuno-precipitation assay (ChIP)

The ChIP Assay Kit was purchased from Beyotime Biotechnology (Shanghai, China). The S11- or DMSO-treated A549/CDDP cells were prepared for ChIP assay, which was performed according to the instructions of the manufacturer. Ac-H4 antibodies were used for immunoprecipitation. OAZ1 promoter primers were used to amplify the DNA isolated by ChIP assay by PCR, and real-time PCR was performed to analyze the amplification product. The sequences of qPCR primers are listed in Supplementary Table 1.

### Mouse xenograft tumors study

To determine the in vivo anti-tumor activity of S11 combined with cisplatin (CDDP), viable NCI-H460/CDDP cells (5 × 10^6^/100 μl PBS per mouse) were subcutaneously injected into the right flank of 7- to 8-week-old male BALB/c nude mice. The group information, treatment process, and measurement procedure are provided in the Supplementary Materials and Methods. The in vivo study was conducted in strict accordance with the recommendations in the Guide for the Care and Use of Laboratory Animals of the National Institutes of Health. The protocol was approved by the Committee on the Ethics of Animal Experiments of the Shenyang Pharmaceutical University.

### Statistical analysis

Differences between experimental groups were evaluated by one-way ANOVA or Turkey’s post-hoc test using the SPSS11.5 software package for Windows (SPSS, Chicago, IL). Survival curves were constructed using the Kaplan–Meier method. Statistical significance was based on a *P*-value of 0.05 (*P* < 0.05, two-tailed test).

## Supplementary information

Supple materials and methods

Supple Figure 1

Supple Figure 2

Supple Figure 3

Supple Figure 4

Supplementary figure legends
