# Supplementary material for: Targeting HDAC/OAZ1 axis with a novel inhibitor effectively reverses cisplatin resistance in non-small cell lung cancer
Source: Cell Death Dis. 2019 May 24;10(6):400. doi: 10.1038/s41419-019-1597-y (PMC6534535; doi:10.1038/s41419-019-1597-y)
Supplement: Supplementary file 1 — Supple materials and methods [file 41419_2019_1597_MOESM1_ESM.docx]

**Materials and Methods**

*1. Patients and chemotherapy*

A total of 101 patients with advanced NSCLC (stage IIIB and stage IV) were enrolled between January 2004 and June 2016 from Wuhan General Hospital of Guangzhou Command (Wuhan, PR, China). The enrolled patients met the following eligibility criteria: histological or cytological confirmation of NSCLC, presence of measurable disease, no adjuvant or neoadjuvant therapy and surgery, no second malignancies, availability of adequate diagnostic tumor tissue (taken from brochoscopic biopsy, percutaneous lung biopsy or metastatic sites). Among 101 patients, included 65 male and 36 female, 15 squamous carcinoma cases and 86 adenocarcinoma cases, and the mean age was 65 years old.

Each patient underwent the treatment with at least two cycles of the first-line platinum–based chemotherapy. Response to treatment was determined after 2-3 cycles by RECIST (Response Evaluation Criteria in Solid Tumors) criteria, which classified the responses into complete response (CR), partial response (PR), stable disease (SD), and progressive disease (PD). SD plus PD were considered as chemotherapy resistance. Overall survival (OS) was defined as the time between the onset of chemotherapy and the date of the last follow up or death from any cause. Ethical oversight and approval was obtained from the Institutional Review Board of Wuhan General Hospital of Guangzhou Command.

*2. Cell viability assay*

The i*n vitro* cell viability was determined by MTT assay. The cells (1×10^5^ cells/ml) were seeded into 96-well culture plates. After overnight incubation, the cells were treated with various concentrations（0.1, 1, 10, 100 μM) of agents for 48 or 72 h. Then 10 μl MTT solution (2.5 mg/ml in PBS) was added to each well, and the plates were incubated for an additional 4 h at 37 °C. After centrifugation (2500 rpm, 10 min), the medium with MTT was aspirated, followed by the addition of 100 μl DMSO. The optical density of each well was measured at 570 nm with a Biotek SynergyTM HT Reader.

*3. HDAC activity assay*

The HDAC assay was conducted with a HDAC ﬂuorescent activity assay kit (Biovison, USA). Briefly, the A549 and A549/CDDP cells were treated with different concentrations of S11 or belinostat (PXD101) for 24 h before assays. Proteins were isolated by using cell lysis buffer (Beyontime, CHN). The protein concentration was measured by BCA protein assay (Beyontime, CHN). The other procedure is same to *in vitro* HDAC assay. The HDAC assay developer (which produces a fluorophore in reaction mixture) was added, and the fluorescence was measured using a microplate reader (Molecular Devices). HDAC activity is presented as the means±SEM of three determinants.

*4. P-gp activity assay*

P-gp activity was determined by Pgp-Glo assay systems (Promega) following the user protocol provided by the manufacturer. Na_3_VO_4_ was used as an ABCB1 ATPase inhibitor. The activity of ABCB1 ATPase was measured in the presence of test compounds incubated with 25 mmol/L recombinant human ABCB1 membranes at 37 °C for 40 minutes. Luminescence was initiated by ATP detection buffer and luminescent activity was immediately read on LB 960 luminometer (Berthold). To rule out the possibility of false positives (unexpected stimulation of ABCB1 ATPase activity by S11), the activity of ABCB1 ATPase in cells receiving different concentrations of S11 plus Na_3_VO_4_ was compared with treatment with Na_3_VO_4_ alone. The differences between the average luminescent signals from Na_3_VO_4_ samples and S11 plus Na_3_VO_4_ samples reflect the luciferase inhibitory values (DRLU) by different concentrations of S11. The difference in luminescent signal between Na_3_VO_4_-treated samples and untreated samples represents the basal ABCB1 ATPase activity. The ABCB1 ATPase activity affected by elacridar was calculated by the difference in luminescent signal between Na_3_VO_4_-treated samples and the adjusted elacridar-treated samples.

*5.* The Rhodamine 123 efflux assay of P-gp function

A549/DDP and NCI-460/DDP cells were incubated with S11 or PXD101 (5 μM) for 24 h. The cells were resuspended in the medium, and then 10 μL/mL Rhodamine 123 staining solution (Santa Cruz Biotechnology, Dallas, TX, USA) was added, and the cells were incubated at 37°C in 5% CO2 for 30 min. The cells were centrifuged at 2000 rpm for 5 min, washed twice with the medium, resuspended, and incubated for 120 min. Next, the cells were centrifuged again, washed twice with PBS, and analyzed by flow cytometry at 488/530 nm. This experiment was repeated three times.

*6. Western blot analysis*

About 1×10^7^ cells were gathered after pre-treatment for the indicated time periods as described previously. Briefly, an equal amount of total protein extracts from cultured cells or tissues were fractionated by 10-15% SDS-PAGE and then electrically transferred onto polyvinylidene difluoride (PVDF) membranes. Mouse or rabbit primary antibodies and appropriate horseradish peroxidase (HRP)-conjugated secondary antibodies were used to detect the designated proteins. The bound secondary antibodies on the PVDF membrane were reacted with ECL detection reagents (Pierce; Rockford, USA) and exposed to X-ray films. Results were normalized to the internal control β-actin.

*7. Scratch-wound healing recovery assays*

Cells seeded in 24-well cell culture plates were transfected at 60–70% confluency in triplicate. Growth medium was removed, and straight incisions were made with a standard 10 μl pipette tip. Cells were washed several times with PBS to remove detached cells. Medium containing 10 ml/l FBS with or without the indicated concentrations of S11and PXD101 were added to the wells and incubated for another 24 hrs. Pictures of the scratches were taken at 0 and 24 hrs. Three representative images of the scratched areas were photographed under a light microscope. Images were acquired with a Leica DMI3000 B Camera System. The wound area was used to quantify the extent of wound healing in each group. The values obtained were expressed as a migration percentage, setting the gap area at 0 hr as 0%.

*8. RNA sequencing and gene expression analysis*

Total RNAs of A549 cells, A549/CDDP cells and A549/CDDP treated with S11 cells were isolated by using RNeasy Mini Kit (Qiagen, Valencia, CA, USA) as described in the product introduction. Array hybridization was performed according to Affymetrix FS450_0002 Hybridization Protocol for gene expression. The Affymetrix GeneChip PrimeView Human Gene Expression Arrays were scanned with Affymetrix Genechip Scanner 7G. For quantitative PCR (qPCR), the RNA was reverse transcribed by using random hexamer primers and a revertAid first-strand cDNA synthesis kit (Invitrogen). qPCR was performed by using iTaq Universal SYBR Green Supermix (Bio-Rad, Hercules, CA, USA). The sequences of qPCR primers are listed in Supplementary Table 1.

*9. Transwell assay*

NCI-H460/CDDP migration capacity was tested by Corning transwell assay, according to the manufacturer’ s instructions. Briefly, the indicated lung cancer cells were treated with Scramble siRNA and OAZ siRNA (100 nM) for 48 h and then seeded in the upper chamber of the system at a density of 5x10^4^ cells/well in serum-free medium (100 μl). The wells in the lower chamber of the system were filled with complete medium. After incubating for 48 h, the cells remaining in the upper chamber were carefully removed with a cotton swab, and the cells that had migrated through the membrane and adhered to its lower surface were fixed with 100% methanol and stained with 0.2% crystal violet. The membrane was then photographed under a microscope, and the cells in five predetermined fields were counted at 200 x magnification.

*10. Mouse xenograft tumors study*

To determine the *in vivo* anti-tumor activity of S11 combined with cisplatin(CDDP), viable NCI-H460/CDDP cells (5×10^6^/100 μl PBS per mouse) were subcutaneously injected into the right flank of 7- to 8- week old male BALB/c nude mice. When the average tumor volume reached 80 mm^3^, the mice were randomly divided into five treatment groups, including control (saline only, n=4), S11(10 mg/kg/3days, i.p.; n=5), S11(20 mg/kg/3days, i.p.; n=5), CDDP (5.5 mg/kg/week, i.p.; n=4), and the combination (S11 10 mg/kg/3days + CDDP 5.5 mg/kg/week, n=4). Tumor size was measured once every three days with a caliper (calculated volume=shortest diameter2×longest diameter/2). Body weight was recorded once every three days. After 21 days, the mice were sacrificed and the tumors were excised and stored at -80°C until further analysis. At the same time, some internal organs (heart, liver, spleen, lung and kidney) were also collected for toxicology study. These studies were performed in strict accordance with the recommendations in the Guide for the Care and Use of Laboratory Animals of the National Institutes of Health. The protocol was approved by the Committee on the Ethics of Animal Experiments of the Shenyang Pharmaceutical University.

*11. Flow-cytometry analysis.*

Analyses for apoptosis were conducted with an Annexin V–FITC Apoptosis Detection Kit (BioVision). Drug resistant cells (1 × 10^6^) were exposed to S11, CDDP, or their combination. They were collected by centrifugation and resuspended in 500 μl of 1 × binding buffer. Annexin V–fluorescein isothiocyanate (FITC; 5 μl) and PI (5 μl) were added to the cells. After incubation at room temperature for 5 min in the dark, cells were analyzed by FACS using a flow cytometer (Becton Dickinson, Bergen, NJ, USA). Cells that stained Annexin V–FITC were analyzed.

**Supplementary table1**

Supple Tab.1 Realtime RT-PCR primer sequence

| Gene | Sequence | |  |
| --- | --- | --- | --- |
|  | Forward | Reverse |  |
| CTGF | CAGCATGGACGTTCGTCTG | AACCACGGTTTGGTCCTTGG | |
| OAZ1 | CTCCACTGCTGTAGTAACCCG | GATCCCTCTGACTATTCCCTCG | |
| β-actin | CTCCATCCTGGCCTCGCTGT | GCTGCTACCTTCACCGTTCC | |
| OAZ1 P1 | ACAGGCGAAACAGTTTACCG | AAAACACTACTCCTGGCAGTT | |
| OAZ1 P2 | AATAATTCTGGCGCGGTTGG | GTTTGTCTGTCGTGGTCCAC | |
| OAZ1 P3 | CAGCGGATCCTCAATAGCCA | ACTTACCTCTCACTGCTGCT | |
