# Supplementary figures and images for: Targeting HDAC/OAZ1 axis with a novel inhibitor effectively reverses cisplatin resistance in non-small cell lung cancer
Source: Cell Death Dis. 2019 May 24;10(6):400. doi: 10.1038/s41419-019-1597-y (PMC6534535; doi:10.1038/s41419-019-1597-y)

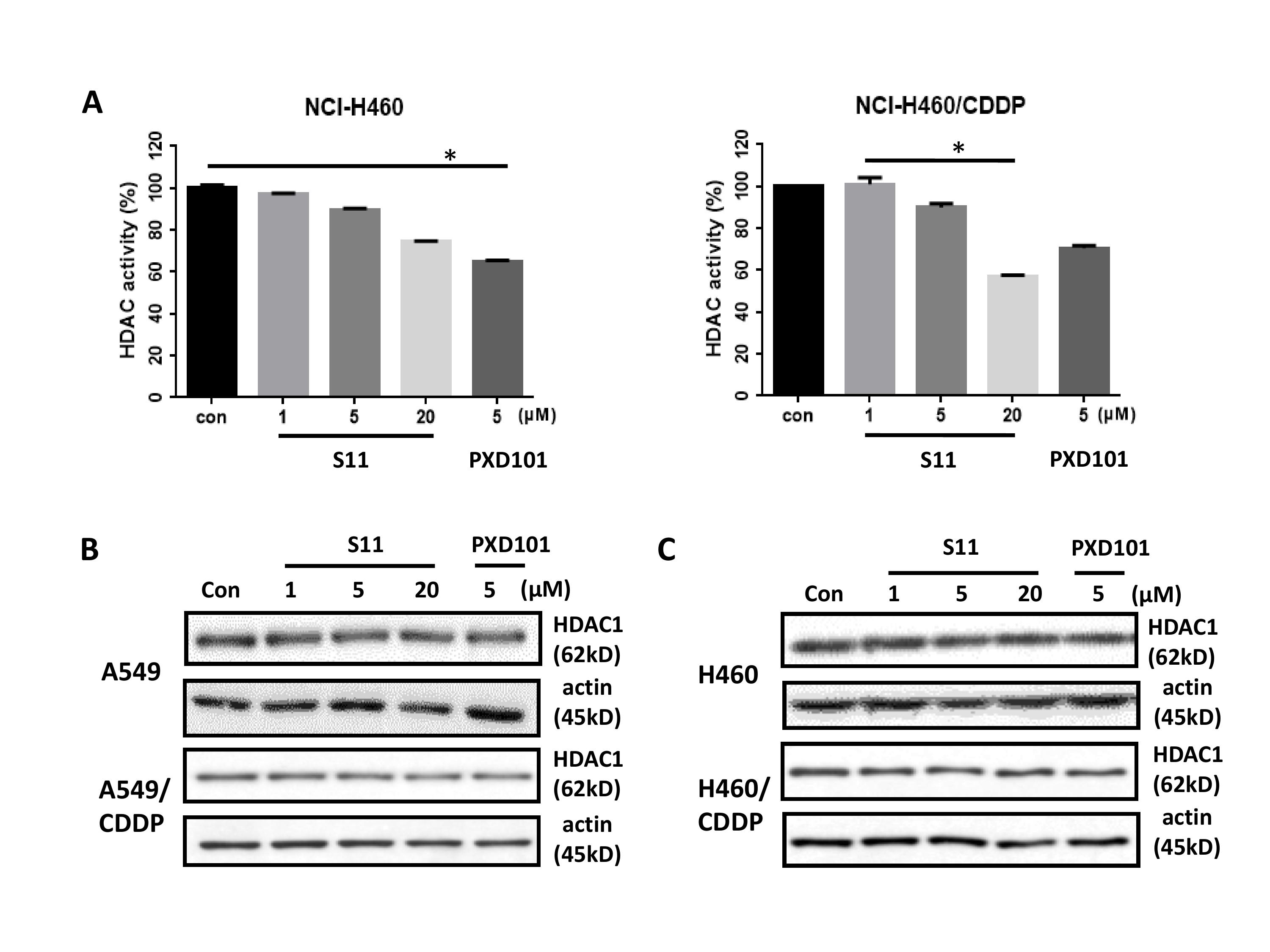

Supplement: Supplementary file 2 — Supple Figure 1 [file 41419_2019_1597_MOESM2_ESM.jpg]

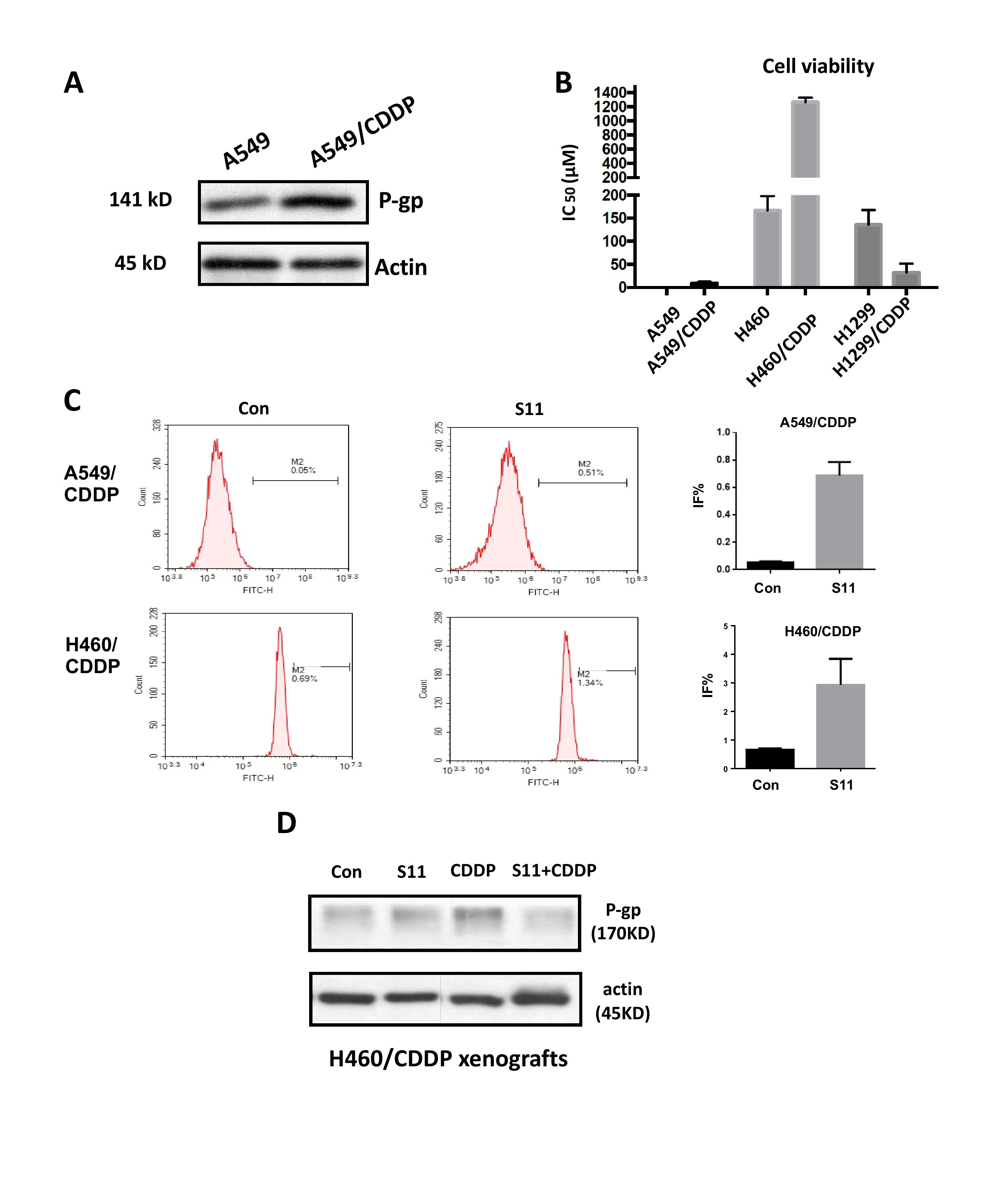

Supplement: Supplementary file 3 — Supple Figure 2 [file 41419_2019_1597_MOESM3_ESM.jpg]

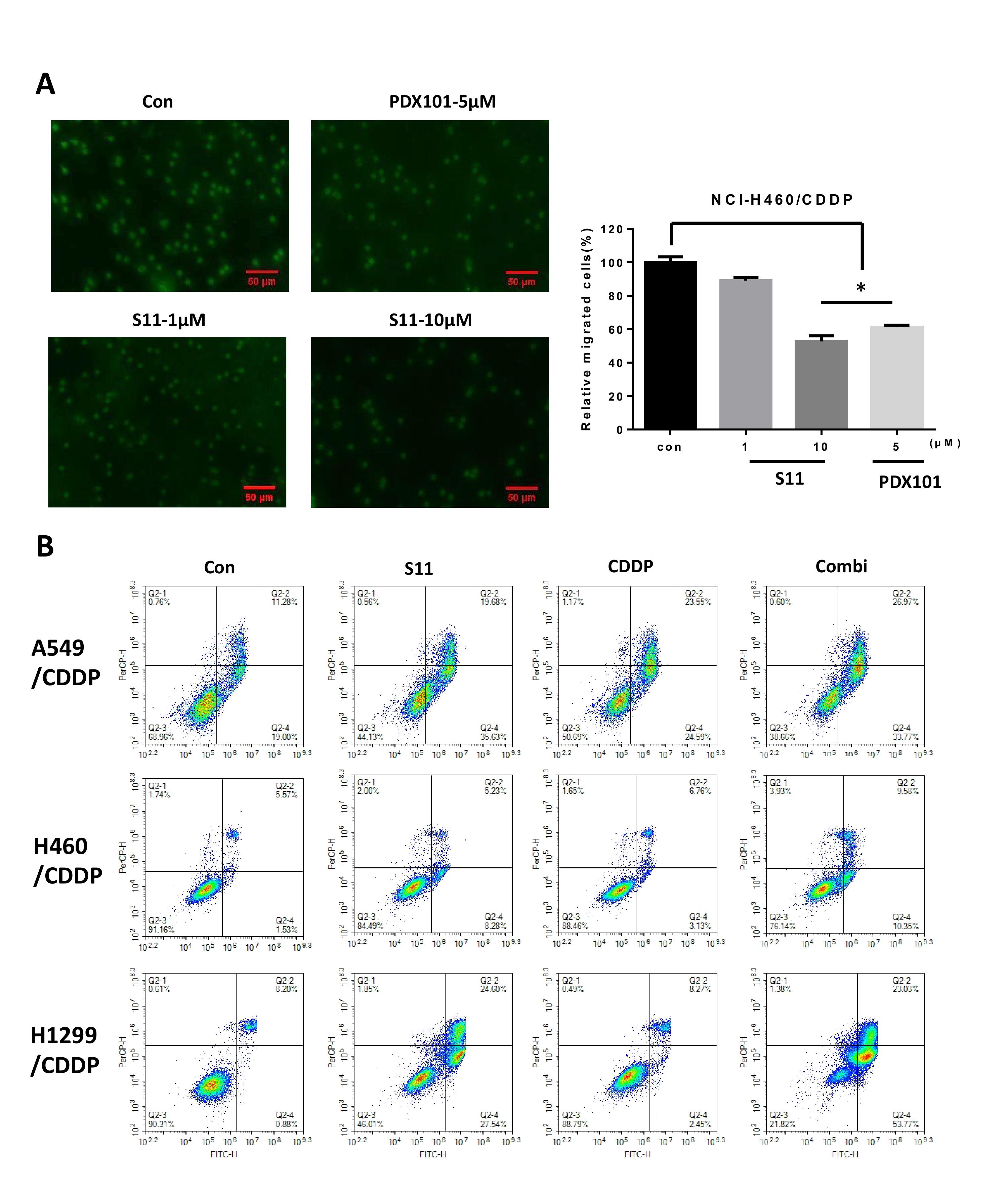

Supplement: Supplementary file 4 — Supple Figure 3 [file 41419_2019_1597_MOESM4_ESM.jpg]

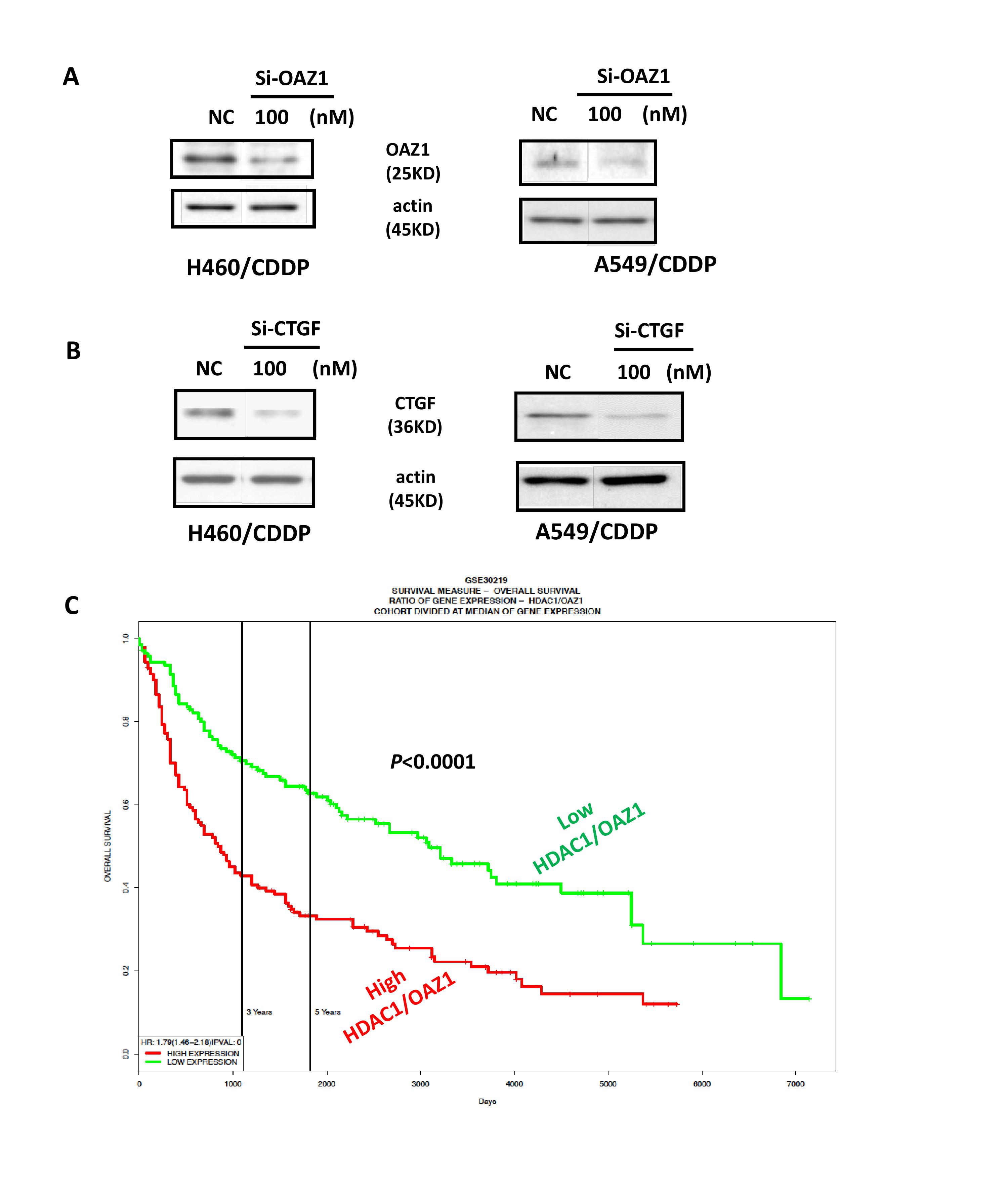

Supplement: Supplementary file 5 — Supple Figure 4 [file 41419_2019_1597_MOESM5_ESM.jpg]
