# Supplementary material for: Targeting HDAC/OAZ1 axis with a novel inhibitor effectively reverses cisplatin resistance in non-small cell lung cancer
Source: Cell Death Dis. 2019 May 24;10(6):400. doi: 10.1038/s41419-019-1597-y (PMC6534535; doi:10.1038/s41419-019-1597-y)
Supplement: Supplementary file 6 — Supplementary figure legends [file 41419_2019_1597_MOESM6_ESM.docx]

**Supplementary Figure 1** (A) HDAC activity was detected by microplate reader in S11 treated and DMSO treated NCI-H460 and NCI-H460/CDDP cells. PXD101 was used as positive control. (B-C) The expression HDAC1 in A549, A549/CDDP, NCI-H460 and NCI-H460/CDDP cells was analyzed after HDAC activity detection.

**Supplementary Figure 2** (A) The expression of P-gp in A549 and A549/CDDP cells. β-actin expression was used as a loading control. (B) The cell viability of A549, A549/CDDP, NCI-H460, NCI-H460/CDDP, NCI-H1299, and NCI-H1299/CDDP cells after treated with different concentrations of PXD101 for 48 h (C) The effects of S11 on intracellular accumulation of Rh123 in A549/CDDP and NCI-H460/CDDP cells were measured by flow cytometry, indirectly reflecting the function of P‑gp following exposure of A549/CDDP and NCI-H460/CDDP cells to S11 or PXD101 for 24 h. (D) The effect of different treatments with S11, CDDP or the combination of S11 and CDDP on P-gp expression in tumor sections from NCI-H460/CDDP xenografts was analyzed by western blot.

**Supplementary Figure 3** (A) The migration ability was accessed in S11 and PXD101 treated NCI-H460/CDDP cells by transwell migration assay. The resultant data were analyzed and graphs from the averaged results of three independent experiments are shown. All error bars are s.e.m. * *P*<0.05, compare with control. (B) The apoptotic cells were assessed by FACS analyses in CDDP resistant NSCLC cells after the treatment with S11, CDDP, or their combination. A549/CDDP Cells were treated with S11 (8.4 μM), CDDP (45.6 μM) or the combination of S11 (8.4 μM) and CDDP (45.6 μM) for 48 h. NCI-H460/CDDP Cells were treated with S11 (38.4 μM), CDDP (25.8 μM) or the combination of S11 (38.4 μM) and CDDP (25.8 μM) for 24 h. H1299/CDDP Cells were treated with S11 (16.6 μM), CDDP (93.1 μM) or the combination of S11 (16.6 μM) and CDDP (93.1 μM) for 48 h.

**Supplementary Figure 4** (A-B) The expression of OAZ1 or CTGF were accessed in A549/CDDP and NCI-H460/CDDP cells after knockdown OAZ1 and CTGF by siRNA for 48 hours. (C) The prognosis significance of HDAC1 along with OAZ1 expression in NSCLCs according to according to data from the PROGgeneV2 database.
